# 

**Published:** 1952-07

**Authors:** 


THE BRISTOL

MEDICO-CHIRURGICAL JOURNAL

*A Journal of the Medical Sciences for the*

WEST OF ENGLAND AND SOUTH WALES

VOL. 69 JULY 1952 NO. 251

### Contents

*PUBLISHED QUARTERLY*

*ANNUAL SUBSCRIPTION 16/- POST FREE*

PUBLISHED UNDER THE AUSPICES OF THE
BRISTOL MEDICO-CHIRURGICAL SOCIETY

### *Editorial Committee*

Mr. H. Keith Lucas, 60 St. John's Road, Bristol 8. *Editor.*
Dr. J. E. Cates, The Dept. of Medicine, Bristol Royal Infirmary. *Assistant Editor.*
Dr. G. E. F. Sutton, 1 Pembroke Road, Bristol 8. *Treasurer.*
Dr. J. H. Middlemiss, 11 Pembroke Road, Bristol 8.
Dr. A. M. MacLachlan, 167 Redland Road, Bristol 6.
Dr. A. H. Gale, The University, Bristol 8.

*Local Correspondents*

*Bath* . . Mr. A. D. Bateman, 3 The Circus, Bath.
*Exeter* . . Dr. L. N. Jackson, Mount Jocelyn, Crediton, Devon.
*Gloucester* . Dr. R. F. Jarrett, 9 College Green, Gloucester.
*Newport* . Mr. J. L. D. Williams, The Knoll, 145 Stow Hill, Newport, Mon.
*Plymouth* . Dr. C. R. Croft, 1 The Crescent, Hartley, near Plymouth.
*Taunton* . Dr. M. McAlley, 1 The Crescent, Taunton.
*Torquay* . Dr. A. Robinson Thomas, 20 Devon Square, Newton Abbot, Devon.
*Truro* . . Dr. F. D. M. Hocking, St. Andrews, Perranporth, Cornwall.
*Yeovil* . . Mr. J. A. Vere Nicholl, Knapp House, Yeovil, Somerset.

### NOTICE TO CONTRIBUTORS

Original articles are invited, provided they have not been published elsewhere. Notes of forthcoming events, meetings held, degrees conferred, staff appointments, and other local news are welcomed. The editorial committee reserves the right to make literary corrections.

Illustrations should always be supplied ready for reproduction. All re-touchings or other operations required will be charged to the author. Line drawings should be drawn in Indian ink. We regret that we must ask authors to pay for making blocks, if this is necessary.

Authors of original articles are entitled to receive twenty-five Reprints, with covers showing source of Reprint, free of charge. Additional reprints may be obtained if notice is given at the time of returning proofs. Approximate cost, with cover as above: 8 pp., 25 copies, 8/-; 50 copies, 12/6. Contributors requiring special covers for their reprints, showing source of reprint with author's name, appointments and title of article, can obtain them as follows: 25 copies, 12/-; 50 copies, 14/6.

J. W. ARROWSMITH LTD., SMALL STREET,
BRISTOL.

### *Appointments*

| *Bristol* | | *Formerly* |
|---|---|---|
| CROSS, E. G. W., M.R.C.S., D.P.M. | Registrar in Mental Health, Bristol Mental Hospitals. | R.A.M.C. |
| EHRMANN, E. H., L.D.S. | Registrar in Dental Surgery, United Bristol Hospitals. | S.H.O. Maxillofacial Unit, Wythenshawe Hospital, Manchester. |
| FOWLER, A. W., M.B., F.R.C.S. | Registrar in Orthopaedics, Winford Orthopaedic Hospital. | Orthopaedic Registrar, Halifax Hospital. |
| FERGUSON, A. W., M.B., M.R.C.P., D.C.H. | Registrar in Paediatrics, United Bristol Hospitals. | Paediatric Registrar, South Warwickshire Hospital Group. |
| GALL, W. J., M.B. | Demonstrator, Department of Anatomy. | |
| HALE, B. T., M.B., D.M.R.T. | Registrar in Radiotherapy, United Bristol Hospitals. | Senior House Officer, Radiotherapy, United Bristol Hospitals. |
| JEANES, E. W., M.B., D.P.H. | Assistant Venerologist, Bristol. | Assistant M.O.H., Rotherham. |
| LUKIANOWICZ, N., M.B., M.D. | Registrar in Mental Health, Bristol Mental Hospitals. | M.O. Polish Rehabilitation Centre, Brighton. |
| MITCHELL, J. P., M.B., F.R.C.S. | Consultant Urologist, Southmead Hospital. | Senior Registrar, Bristol United Hospitals. |
| PEACOCK, J. H., M.B., F.R.C.S. | Lecturer in Surgery, University of Bristol. | Rockefeller Scholar, U.S.A. |
| PENGELLY, C. D. R., M.B. | Registrar in Medicine, United Bristol Hospitals. | Senior House Officer, Glasgow Royal Infirmary. |
| RICHARDS, F. B., M.B. | Assistant Physician in Chest Diseases, Bristol. | Senior Registrar, Chest Diseases, Bristol. |
| STEEN, T. R., M.B., D.A. | Consultant Anaesthetist, Bristol. | Senior Registrar, United Bristol Hospitals. |
| TAYLOR, A. W., M.B. | Registrar in Mental Health, Bristol Mental Hospitals. | R.A.M.C. |
| WOOD, F. J. Y., M.B. | Registrar in Medicine, United Bristol Hospitals. | Travelling Scholarship, U.S.A. |

| | | Formerly |
|---|---|---|
| *Bath* | | |
| BRUCE-LOCKHART, P., M.B. | Registrar in Obstetrics and Gynaecology, Bath Group. | Re-appointment. |
| LONDON, P. S., M.B., F.R.C.S. | Senior Registrar in Orthopaedic and Traumatic Surgery, Bath. | Registrar, Birmingham Accident Hospital. |
| *Gloucestershire* | | |
| DANCE, J. M., M.B., D.C.H. | Registrar in General Medicine, Gloucestershire Royal Infirmary. | Senior House Physician, Gloucestershire Royal Infirmary. |
| HACZKIEWICZ, S., M.D. | Assistant Physician in Chest Diseases, North Gloucestershire. | Junior Hospital Medical Officer, Standish House Sanatorium, Stonehouse. |
| *Cornwall* | | |
| JACKSON, E. G. A., M.B. | Registrar in Anaesthetics, South Devon and East Cornwall Hospital. | Senior House Officer, Royal Devon and Exeter Hospital. |
| JOLLY, H. R., M.B., M.R.C.P., D.C.H. | Consultant Paediatrician, Plymouth. | Senior Registrar, Hospital for Sick Children, Great Ormond Street. |

